# Recurrent Pericarditis With Cardiac Tamponade in a Patient With Systemic Lupus Erythematosus: A Case Report

**DOI:** 10.7759/cureus.116182

**Published:** 2026-09-13

**Authors:** Sami Khatib, Chirag Lodha, Eric J Basile

**Affiliations:** 1 Internal Medicine, University of South Florida Morsani College of Medicine, Tampa, USA; 2 Cardiovascular Disease, University of South Florida Morsani College of Medicine, Tampa, USA

**Keywords:** cardiac tamponade, lupus pericarditis, pericardial window, pleuropericarditis, recurrent pericardial effusion, systemic lupus erythematosus

## Abstract

A 29-year-old male with a history of systemic lupus erythematosus (SLE) with class IV/V lupus nephritis, recurrent pericardial effusions, hypertension, and type 2 diabetes mellitus presented for worsening chest tightness and shortness of breath. Echocardiography revealed a large, free-flowing pericardial effusion circumferential to the heart, up to 2.9 cm at its widest diameter, with mild atrial and right ventricular collapse. Due to concern for tamponade, interventional cardiology was consulted, and a pericardiocentesis was performed, yielding 820 mL of serosanguinous fluid, and a pericardial drain was kept in place. Given his history of SLE, immunosuppressive therapy was started with intravenous methylprednisolone pulse dosing 250 mg twice a day for three days, followed by an oral prednisone taper and mycophenolate mofetil 500 mg twice a day titrating up to 1500 mg twice a day, as well as increasing hydroxychloroquine to 400 mg daily from 200 mg daily, starting colchicine 0.6 mg daily, and continuing voclosporin 23.7 mg twice a day. However, his pericardial effusion persisted, ultimately requiring a pericardial window. Unfortunately, just a few weeks later, he returned again with recurrent pleuropericarditis in spite of aggressive medical and surgical management and ultimately elected to leave against medical advice. This case highlights the challenges in managing recurrent pericardial effusions in patients with SLE.

## Introduction

Pericarditis is a common cause of chest pain and shortness of breath, leading to emergency department visits and hospital admissions with a broad differential, including infectious, autoimmune, neoplastic, traumatic, and metabolic causes [1]. In up to 60% of cases, there is an associated pericardial effusion [1,2]. Pericardiocentesis and pericardial fluid analysis can be performed to aid with diagnosis, but the utility varies depending on the underlying etiology. For example, adenosine deaminase levels have been shown to be highly sensitive and specific (90% and 86%, respectively) for tuberculous pericarditis, and cytology is often valuable in the diagnosis of malignant effusions [2,3]. However, the diagnosis of non-malignant and non-infectious effusions can be more challenging as pericardial fluid biochemistry is generally of limited value in diagnosing the underlying etiology [3]. Furthermore, Light’s criteria, which are often used to classify pleural effusions as transudative or exudative, are unreliable for pericardial effusions as Light’s criteria have been shown to misclassify even normal pericardial fluid as exudative [4]. Thus, a thorough diagnostic workup and evaluation of the overall clinical picture is necessary for diagnosis of the underlying etiology of pericarditis and pericardial effusions.

Pericarditis with an associated pericardial effusion is a common complication in patients with systemic lupus erythematosus (SLE), affecting approximately 20% of patients [5]. In fact, acute pericarditis is one of the criteria for the diagnosis of lupus in the current gold standard diagnostic guidelines from the 2019 European League Against Rheumatism/American College of Rheumatology (EULAR/ACR) classification criteria, though it requires a thorough workup to ensure sufficient criteria for diagnosis are met and other potential etiologies are excluded [6]. In most cases, the effusion resolves with treatment of the underlying SLE with anti-inflammatory and disease-modifying agents, with only about 22% of these patients experiencing relapses [5]. Furthermore, progression to tamponade is rare but potentially life-threatening, occurring in about 1.4% of SLE patients with pericardial effusions [5]. Tamponade in these patients is generally managed with urgent pericardiocentesis along with anti-inflammatory and disease-modifying agents, but, in refractory cases, a pericardial window may be necessary [7,8]. We present a case of pericarditis with recurrent pericardial effusions causing cardiac tamponade in a patient with SLE that persisted despite pericardiocentesis and medical management, ultimately requiring a surgical pericardial window.

## Case presentation

This patient is a 29-year-old male with a history of SLE with class IV/V lupus nephritis and recurrent pericardial effusion requiring prior pericardiocentesis and drain placement, hypertension, and type 2 diabetes mellitus who was admitted with recurrent pericardial effusion presenting as progressive shortness of breath and chest tightness, similar to his symptoms with prior episodes. Physical exam was notable for hypertension to 182/117 and tachycardia to 117 bpm but otherwise relatively unremarkable, as he was breathing comfortably with an oxygen saturation of 99% on room air without any pericardial friction rub, distant heart sounds, or signs of volume overload such as jugular venous distension noted.

On admission, echocardiography demonstrated a large, free-flowing pericardial effusion circumferential to the heart, measuring up to 2.9 cm, with mild right atrial and right ventricular collapse concerning for early tamponade physiology, as well as a left-sided pleural effusion (Figure 1). Chest X-ray confirmed cardiomegaly and a small-to-moderate left pleural effusion with left basilar atelectasis (Figure 2).

**Figure 1:**
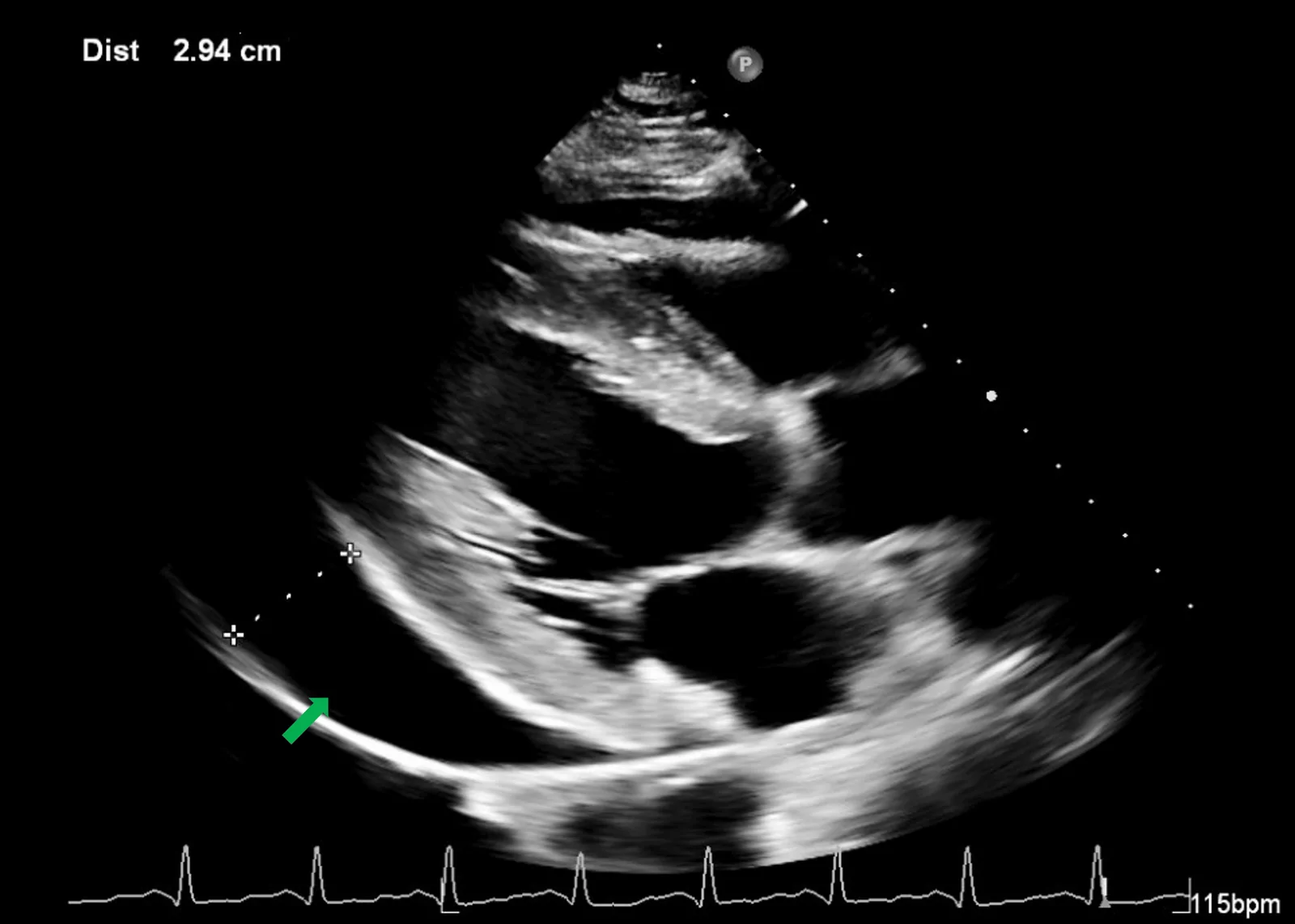
Echocardiography showing a large pericardial effusion. Echocardiography on admission showing a large pericardial effusion measuring up to 2.94 cm (as denoted by the green arrow).

**Figure 2:**
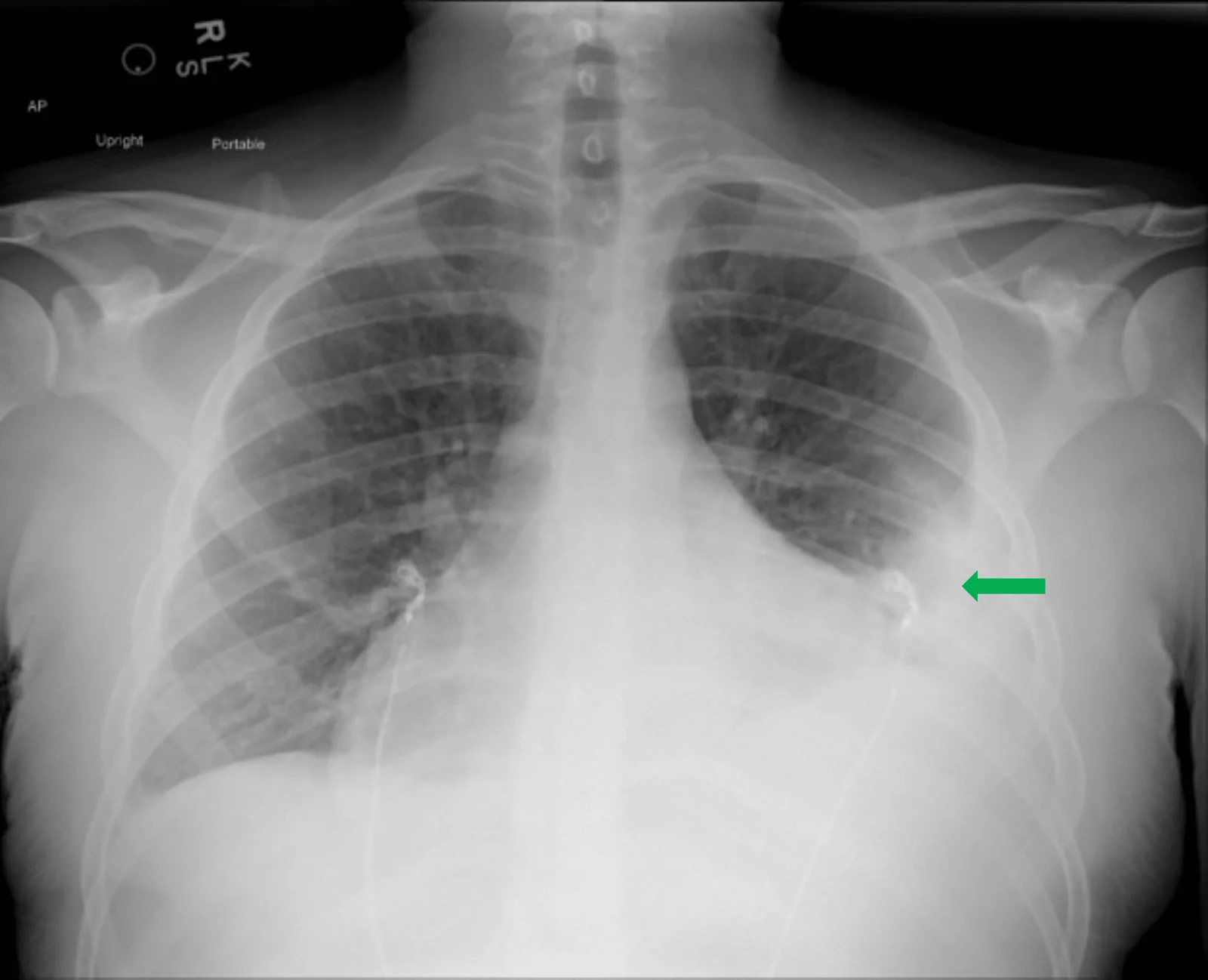
Chest X-ray showing a left pleural effusion. Chest X-ray on admission showing a small-to-moderate left pleural effusion (as denoted by the green arrow) as well as cardiomegaly and left basilar atelectasis.

Cardiothoracic surgery, cardiology, rheumatology, and nephrology were consulted. Initial recommendations included pericardiocentesis for the large effusion given the concern for tamponade, with rheumatology input for optimization of immunosuppression for SLE-related serositis. After multidisciplinary discussion, the patient underwent echo-guided subxiphoid pericardiocentesis with placement of a pericardial drain, yielding 820 mL of serosanguinous fluid, followed by continued drainage and monitoring. Pericardial fluid studies were sent, and the drain was managed to gravity with removal planned once output was less than 50 mL over 24 hours. Biochemistry studies of the fluid showed protein 5.6 g/dL, albumin 2.0 g/dL, lactate dehydrogenase 186 U/L, glucose 105 mg/dL, and pH 7.98 with serum protein 8.2 g/dL (N = 6.4-8.3 g/dL), albumin 2.6 g/dL (N = 3.5 - 5.0 g/dL), and glucose 102 mg/dL (N = 70-110 mg/dL). Furthermore, cytology showed 40,000 RBCs/µL and 307 nucleated cells/µL, with a differential showing 5% neutrophils, 9% lymphocytes, 72% monocytes/macrophages, 1% eosinophils, 3% mesothelial cells, 10% other cells, and no malignant cells. Cultures, including anaerobic cultures, were negative. Thus, the lack of malignant cells and no history of active malignancy lowered concern for a malignant effusion, and the negative cultures, lack of neutrophilic predominance, and lack of other infectious symptoms lowered concern for a bacterial effusion. In addition, the serum blood urea nitrogen (BUN) was normal (12, N = 6-20 mg/dL), making uremic pericarditis unlikely.

Renal biopsy from about a year prior had shown diffuse proliferative glomerulonephritis with full house immune complex deposition and membranous glomerulopathy consistent with diffuse lupus nephritis, International Society of Nephrology and Renal Pathology Society (ISN/RPS) class IV, and membranous lupus nephritis, ISN/RPS class V. Screening for potential infectious causes, including hepatitis B and C, HIV, and tuberculosis (TB), was negative, and the patient had no history of long-term use of medications such as hydralazine or procainamide that can cause drug-induced lupus. Other serum studies showed positive antinuclear antibodies with high titers 1:5120, positive double-stranded DNA antibodies, positive anti-Smith antibodies, and hypocomplementemia with C3 34 mg/dL (N = 82-173 mg/dL) and C4 < 2.9 mg/dL (N = 15.0-53.0 mg/dL), as well as a urine protein-creatinine ratio of 3.8 (N < 0.2) and serum creatinine of 1.3 mg/dL (N = 0.6-1.2 mg/dL), all consistent with lupus nephritis.

Given the recurrent nature of his effusion and underlying active lupus nephritis, immunosuppressive therapy was escalated in coordination with rheumatology and nephrology. He was started on intravenous methylprednisolone pulse dosing with 250 mg twice daily for three days, followed by oral prednisone 40 mg daily with a taper, as well as trimethoprim/sulfamethoxazole 800/160 mg every other day for *Pneumocystis jirovecii* pneumonia prophylaxis and lansoprazole 30 mg daily for gastrointestinal ulcer prophylaxis. We also added oral mycophenolate mofetil 500 mg twice a day, titrating up to 1500 mg twice a day; increased the dose of hydroxychloroquine to 400 mg daily from 200 mg daily; initiated colchicine 0.6 mg daily; and continued voclosporin 23.7 mg twice a day. Lisinopril was continued for proteinuria and hypertension, and additional antihypertensive therapy was considered as tolerated.

Following the pericardiocentesis, the patient remained hemodynamically stable and reported improvement in symptoms. However, the drain output continued to be elevated at >50 mL/24 hours for over a week. Because of this and the recurrent nature of the effusion in spite of immunosuppressive therapy, he was taken for left robotic thoracoscopic creation of a pericardial window by cardiothoracic surgery with exchange of the left pericardial drain for a large-caliber drain. Surgical pathology showed thickened fibrovascular connective tissue and fibroadipose tissue with nonspecific lymphoplasmacytic inflammation and focal reactive mesothelial proliferation without evidence of malignancy. His condition improved over the next few days, and he was stable for removal of the drain and discharge a few days later.

During the admission, he also underwent ultrasound-guided thoracentesis for the left pleural effusion, with removal of 1,300 mL of clear yellow fluid without complication. He did briefly develop an acute kidney injury requiring holding voclosporin, but it resolved within a few days with return of his creatinine to normal levels. Nephrotic-range proteinuria persisted, consistent with active lupus nephritis that was managed with immunosuppressive therapy escalation as described above. Anemia was noted but remained stable without requiring any acute intervention prior to discharge.

Unfortunately, he was re-admitted a few weeks later due to worsening shortness of breath with recurrent pericardial and pleural effusions requiring left chest tube placement as well as continued aggressive immunosuppressive therapy. Cardiothoracic surgery recommended exhausting all medical options before considering a repeat pericardial window. The pleural effusion had begun slowly improving, but the chest tube still had high output, and there was a persistent moderate-to-large pericardial effusion without evidence of tamponade physiology. As part of a multidisciplinary discussion, rheumatology recommended a trial of anakinra 100 mg daily while inpatient due to his refractory pleuropericarditis, which he was started on. However, before further management steps could be performed, the patient decided to leave against medical advice five days after admission with his chest tube still in place due to social factors, including family matters and financial concerns, despite counseling about the severity of his condition. It was determined that he had the capacity to make this decision. Therefore, our ability to assess treatment effectiveness, particularly of the recently initiated biologic therapy, is limited.

## Discussion

Pericarditis, as well as other forms of serositis, commonly occurs in patients with SLE. In a recent cohort study of 2,122 SLE patients, Mehta et al. found that pericarditis occurred in about 20% of lupus patients [5]. While outcomes were generally favorable, 22.9% of patients experienced relapses [5]. Interestingly, age at diagnosis, organ involvement, and serology were not found to be associated with relapse risk, making it difficult for clinicians to predict which patients may be at increased risk for relapse [5]. While methotrexate and biologics use was associated with relapse, this association may be due to confounding since these drugs are more likely to be used as second- or third-line therapy [5].

Tamponade is a very rare complication of lupus pericarditis, occurring in only 1.4% of patients [5]. Unfortunately, this means there is limited data on the treatment and prognosis of tamponade in lupus pericarditis. There have been some case studies in which the tamponade was successfully managed acutely with urgent pericardiocentesis and anti-inflammatory and disease-modifying agents, but refractory cases required surgical intervention with a pericardial window [7,8]. In a retrospective study of 409 lupus patients, Goswami et al. found that larger effusion size, associated pleuritis, and positive serum anti-nucleosome antibody titers were all associated with an increased risk of tamponade [9]. In addition, while a recent retrospective cohort study found that oral corticosteroid use may increase the risk of recurrent lupus pericarditis, the 2025 ACR guidelines for the treatment of SLE conditionally recommend treating organ- or life-threatening SLE flares with intravenous methylprednisolone pulse dosing followed by an oral glucocorticoid taper [10,11]. Thus, we elected to treat with corticosteroids in combination with other immunosuppressive therapies given the presence of tamponade. However, there is very limited data on the long-term prognosis, risk of recurrence, and management of recurrence in these patients.

There are a number of biologic therapies that have been developed for the treatment of SLE that may provide benefit in refractory cases such as this one. Rituximab, targeting CD20 on B cells, was one of the first to become available [12]. More recently, there are a variety of other biologics that have been developed. Belimumab also targets B cells, specifically by inhibiting B-cell activating factor [12]. Furthermore, anifrolumab inhibits the type 1 interferon receptor and has been approved for treatment of SLE. Finally, interleukin-1 blocking agents have also been used in some cases of refractory lupus, including anakinra, a recombinant interleukin-1 receptor antagonist, and rilonacept, an interleukin-1 decoy receptor [13].

While pericardial effusions are initially managed medically in stable patients, procedural or surgical intervention may be indicated in refractory cases or cases with tamponade physiology on imaging [14]. Although pericardiocentesis is typically first-line because it is less invasive, it has higher recurrence rates than pericardial windows, which is why we decided to proceed with a pericardial window in this patient with recurrent pericardial effusions in spite of pericardiocentesis and drain placement [14]. Most of the research on outcomes in patients who underwent pericardial window formation has been done in cases of pericardial effusions in patients with underlying cancer rather than SLE. In a study including 48 cancer patients with pericardial tamponade treated with a pericardial window, Celik et al. found no surgery-related mortality, a 30-day post-operative mortality rate of 8.33%, a median overall survival of 10.41 months with a mean follow-up duration of 38.86 months, and a symptomatic recurrence rate of about 2% (only one patient) [15]. In a larger study including 166 cancer patients with pericardial effusions treated by pericardial window creation, Kim et al. found no surgery-related mortality and a one-year overall survival rate of 31.4%, with only 9.6% of patients requiring repeat intervention due to recurrence [16]. While one-year survival rates were higher in patients who received thoracoscopic surgery than those who received thoracotomy, this may have been due to selection bias, as patients with poorer prognoses are more likely to receive thoracotomy, and there was no difference in recurrence rates [16].

## Conclusions

In conclusion, pericarditis with an associated pericardial effusion is a common complication of SLE, but recurrent effusions and development of cardiac tamponade are rarer and more serious complications. While pericardial effusions can often be managed medically, cardiac tamponade is a medical emergency that requires immediate procedural intervention. When pericardiocentesis and medical optimization fail, pericardial window creation is a more invasive option that has been shown to have lower recurrence rates. More recently, the development of newer biologic therapies has provided another option for treating challenging cases of recurrent pleuropericarditis.
